# Decomposition, nitrogen and carbon mineralization from food and cover crop residues in the central plateau of Haiti

**DOI:** 10.1186/s40064-016-2651-1

**Published:** 2016-07-04

**Authors:** M. J. Lynch, M. J. Mulvaney, S. C. Hodges, T. L. Thompson, W. E. Thomason

**Affiliations:** Department of Crop and Soil Environmental Science, Virginia Tech, Blacksburg, VA 24061 USA; West Florida Research and Education Center, University of Florida, Jay, FL 32565 USA

**Keywords:** Conservation agriculture, Cover crop, Haiti, No tillage, Nutrient cycling

## Abstract

Cover crops are a major focus of conservation agriculture efforts because they can provide soil cover and increase nutrient availability after their mineralization in cropping systems. To evaluate the effect of residue type and placement on rate of decomposition and carbon (C) and nitrogen (N) mineralization, residues from two food crops, maize (*Zea mays* L.) and common bean (*Phaseolus vulgaris* L.), and two promising cover crops, sunn hemp (*Crotalaria juncea* L.) and sorghum sudangrass (*Sorghum bicolor* [L.] Moench *x S. bicolor* var. *Sudanese* [Piper] Stapf) were used in a litterbag study in the Central Plateau region of Haiti from May to September, 2013. Residues were placed in litterbags at a rate equivalent to 3.25 Mg residue ha^−1^ either on the soil surface or buried at 15 cm to represent a tilled and no-tillage system, respectively. Initial C:N ratios were: maize > common bean > sorghum sudangrass > sunn hemp. Highest residue mass loss rates and C and N mineralization generally occurred in the reverse order. Overall, surface-placed residues decomposed more slowly with 40 and 17 % of initial residue mass of surface and buried residues, respectively, remaining at 112 days. Carbon and N mineralization was higher when residues were buried. Net N mineralization of buried residues was 0.12, 0.07, 0.06, and 0.03 g N g residue^−1^ for sunn hemp, sorghum sudangrass, maize, and common bean, respectively over 112 days. To achieve the goal of increasing nutrient supply while maintaining year-round cover, a combination of grass and legume cover crops may be required with benefits increasing over multiple seasons.

## Background

In Haiti, the poorest country in the Western Hemisphere, 60 % of Haitian farmers have less than one hectare while less than 4 % farm more than five hectares (Cohen 2010). This pressure on landholdings has resulted in an overall expansion of hillside agriculture production on steep slopes (McClintock 2004; MARDNR 2010). Less than 1 % of original forests remain in Haiti (White and Jickling 1995) and soil fertility is generally depleted. Nearly 85 % of land in Haiti is classified as “greatly damaged or in the process of rapid destruction” (Voigt et al. 2011). Plowing and burning are the most prevalent land clearing practices (N. Kennedy, personal communication 2012; McClintock 2004) and contribute to the estimated 36.5 million tons of soil lost annually (Badrie 2007). As in many smallholder farming systems, crop residues are important sources of livestock feed, though because of the limited number of large ruminants, there is less pressure on this resource (McClintock 2004). Farmers in Haiti do not typically gather residues but let animals graze fields after harvest and then burn any residue or vegetation in the field prior to plowing.

The Food and Agriculture Organization of the United Nations (FAO) defines conservation agriculture (CA) as: continuous minimum mechanical soil disturbance, permanent organic soil cover, and diversification of crop species grown in sequences and/or associations (FAO 2011). The use of cover crops and reduced tillage has repeatedly demonstrated increased nutrient mineralization (Mulvaney et al. 2010; Perin et al. 2006), increased accumulation of SOM (Bessam and Mrabet 2003; Ogle et al. 2012; Salinas-Garcia et al. 1997), and reduced erosion (Franzluebbers 2002; Roth et al. 1988). The use of crop residues for livestock feed may hamper the adoption of CA practices in Haiti as it has in other places, therefore the introduction of cover crops may prove especially important as a method to increase SOM.

Rotations of crop mixtures containing grass residues with high C:N ratios and legumes with low C:N ratios can contribute N to the following crop via decomposition of residues left as soil cover after harvest (Abera et al. 2012; Karpenstein-Machan and Stuelpnagel 2000a, b; Perin et al. 2006). Cover crops such as sunn hemp (*Crotalaria juncea* L.) can produce biomass of up to 6 Mg ha^−1^ of dry biomass, and 150–165 kg ha^−1^ of fixed N under favorable conditions in 60–90 days (Rotar and Joy 1983; Clark 2007). Moreover, sunn hemp decomposes quickly. Peak mineralization rates occurred at 2 weeks after burial in a litterbag study conducted on a sandy soil in Florida, USA and also within 2 weeks after initiation of a litterbag study in a sandy loam soil in Ghana (Wang et al. 2007; Fosu et al. 2007). Researchers in Alabama, USA placed litterbags containing sunn hemp in both a sandy soil and a fine sandy loam soil and reported higher maize (*Zea mays* L.) yields with sunn hemp and that sunn hemp generally provided more than adequate biomass coverage to achieve erosion control (Balkcom and Reeves 2005).

Ibewiro et al. (2000) suggested that C:N ratio and mass loss rates of surface litter can be used as indicators of N release and synchronization of N release with crop demand. Two legume and one cereal cover crop were used to measure N fixation and release over 2 years in a sandy loam soil of a savanna in tropical Nigeria. Legume residues with lower C: N ratios resulted in increased dry matter yield and N uptake by the following crop while decomposing faster than the cereal residues (Ibewiro et al. 2000). Abera et al. (2014) conducted a litterbag study of legume residue decomposition in tropical Sub-Saharan Africa and also observed rapid decomposition rates for legumes. They found that 89 % of the initial N in pigeon pea (*Cajanus cajan* L.) and 85 % of N in haricot bean (*Phaseolus vulgaris* L.) were released 150 days after litterbag installation. Relative to the control, leaving legume residue in place resulted in two and threefold increases in following crop maize grain yield.

Murungu et al. (2011) measured N and P mineralization rates of two legume and two cereal crops and found that legume cover crop residues resulted in higher N contribution to following crops than the cereal residue. Nitrogen release from decomposing legume cover crops and their effect on maize yields were measured in the Bukoba District of Tanzania and adding legume cover crops resulted in a twofold maize grain yield increase in a high rainfall area and a three-fold increase in an area of low rainfall (Baijukya et al. 2006). Cookson et al. (1998) installed a litterbag decomposition study on a silt loam soil in New Zealand comparing two cereal crops with a legume and report that mass loss from legume residue was nearly twice that of the cereal crop after 90 days of decomposition. Heal et al. (1997) report that N mineralization and litter decomposition is rapid when residue C:N ratio is less than 20. Therefore litter with low C:N ratio is expected to decompose more rapidly than litter with a higher C:N ratio. This rapid decomposition and N release from legume residue can result in increased grain yield of the following crop when conditions favor good yields.

Many researchers have reported that lignin and soluble polyphenol content, along with C and N content influence residue decomposition and nutrient cycling (Clement et al. 1995; Constantinides and Fownes 1994; Fox et al. 1990; Matta-Machado et al. 1994; Palm and Sanchez 1991; Palm et al. 2001). In fact Palm et al. (2001) propose criteria for N, polyphenol and lignin content that allow residue to be placed into four quality classes. Similarly the plant residue quality index (PRQI), calculated based on residue C:N ratio, lignin and polyphenol was proposed by Tian et al. (1995). In this index, a higher PRQI value generally indicates higher residue quality, greater N mineralization potential and faster decomposition. Previous studies have reported lignin content in maize stover to range from 6.8 to 0.57 % and soluble phenolic compound concentration from less than 1 % (Muhammad et al. 2011; Sakala et al. 2000; Tian et al. 1992, 1995). Lignin and polyphenol levels in *Sorghum bicolor* were 8.1 and 0.55 % respectively when grown in an alley-cropping system in Georgia, USA (Matta-Machado et al. 1994). In fact, Tian et al. (1995) found relatively low levels of lignin maize stover; 6.8 % in compared to 17.5 % for leaves of various leguminous trees, and polyphenols; 0.56 for maize and 3.96 % for legume tree leaves. Similarly, relatively low levels of soluble polyphenol content for *Crotalaria spp.* have been reported and range from 0.8 to 1.2 % (Baijukya et al. 2006; Ndufa 2001) while lignin levels ranged from 4.6 to 8.1 % (Baijukya et al. 2006; Millar and Baggs 2004).

The MARDNR (2010) notes that Haitian farmers are willing to invest in new technologies when the demonstrated economic benefits of incorporation are observable and risks minimal. However, barriers to the adoption of CA principles and the incorporation of cover crops in the Central Plateau are certainly prevalent. The implementation of cover crops such as sunn hemp and sorghum sudangrass hybrids could save the farmer labor expenditures in tillage; however, the cost of seeds may be a deterrent and the overall value of N and other nutrient supply from residue is unknown. Also of concern is the need to dedicate limited arable land to the production of a non-edible cover crop that offers no nutritional or immediate economic value. Quantification of some of the potential benefits to the overall system is essential to increasing adoption of cover crops. Therefore this study set out to document decomposition and mineralization rates for potential cover crops, sunn hemp and sorghum sudangrass and common cash crops, common bean and maize grown in the Central Plateau.

## Methods

### Site description

A field experiment was initiated at the Centre de Formation Fritz Lafontant near Corporant, Haiti (18°55′N, 72°07′W, 158.5 m above mean sea level) in May, 2013. Initial soil physical and chemical parameters (0–15 cm) for the site are presented in Table 1 and initial plant residue C and N content and C:N ratio are shown in Table 2. Soil pH was determined using a 1:1 (vol/vol) soil–water mix (Maguire and Heckendorn 2011). Due to the calcareous nature of the soil, an Olsen extraction method utilizing sodium bicarbonate extractant for available phosphorous was utilized (Olsen et al. 1954) and analyzed using inductively coupled plasma atomic emission spectroscopy (ICP). For other nutrients, soil was extracted with Mehlich 1 at a 1:1 solution:soil ratio (Mehlich 1976) filtered (Whatman #2), and analyzed via ICP (SPECTRO ARCOS, Kleve, Germany). Soil organic matter was estimated using loss on ignition (LOI) (Blue M Ultra-Temp, Blue M, White Deer, PA) paired with ICP where a gravimetric, dry oxidation method was used to estimate the soil organic matter content for all samples (Davies 1974; Pansu and Gautheyrou 2007). Estimated sum of cations was determined using Mehlich 1 extractable bases, or non-acid generating cations (Ca, Mg and K), plus the acidity estimated from the Mehlich soil-buffer pH after conversion of all analytical results to meq/100 cm^3^ or cmol(+)/kg (Maguire and Heckendorn 2011). Soil texture was determined via the hydrometer method. Plant tissue C and N concentrations were determined by dry combustion (VarioMax CNS macro elemental analyzer, Elementar, Hanau, Germany).

**Table 1:** Initial physical and chemical parameters for soil (0–15 cm) at the Corporant experimental site, 2013

pH^a^	7.4
NO3-N^b^ (mg/kg)	2.1
NH4-N^b^ (mg/kg)	1.8
Total N (mg/kg)	1412
Phosphorus^c^ (mg/kg)	12
Potassium^d^ (mg/kg)	87
Magnesium^d^ (mg/kg)	328
Calcium^d^ (mg/kg)	5438
Cation Exchange^d^ (meq/100 g)	30
Organic Matter^e^ (%)	1.6
Sand^f^ (%)	41
Silt^f^ (%)	38
Clay^f^ (%)	21

^a^1:1 soil water

^b^NH4-N and NO3-N: 2 M KCL; automated flow injection analysis

^c^NaHCO3 extraction

^d^Mehlich 1 extraction

^e^Loss on ignition

^f^Hydrometer method

**Table 2:** Initial nitrogen (N) and carbon (C) concentrations and C:N ratio for plant residues used in the litterbag decomposition study

Residue	N (%)	C (%)	C:N
Maize	0.65	36.7	56.4
Sorghum sudangrass	1.09	34.3	31.4
Common bean^a^	0.64	34.8	54.4
Sunn hemp	1.23	36.9	30.0

^a^Common bean residue was weathered post-harvest

**Fig. 1:**
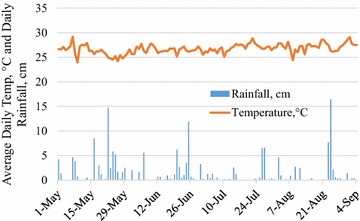
Daily average temperature and cumulative rainfall near Corporant, Haiti, May to September, 2013

Average annual climate for the Central Plateau, where Corporant is located is humid subtropical with large local variations due to the mountainous topography and reported annual precipitation is 1016–1524 mm (Erlich et al. 1987). Rainfall in the region is bimodal with the main rainy season occurring in May–June and a second season in August (McClintock 2004). The dry season stretches from October to March (McClintock 2004). Most crops are rainfed with sowing in May after the first rains. Some fields are planted to a second crop in August depending on the seasonality of crops in rotation (McClintock 2004). Temperature and precipitation measured using an on-site weather station (Watchdog 2000, Spectrum Technologies, Aurora, IL) from the study period are reported in Fig. 1.

### Treatments

Plant residues from the study were collected in February, 2013, from fields near the Centre de Formation Fritz Lafontant near Corporant. Maize and common bean residues were collected after the most recent harvest while sunn hemp and sorghum sudangrass were collected at full bloom/heading. After field collection, all residues were individually placed in a solar drier until a constant weight was reached and stored on site prior to further preparation.

In May, 2013, residues were placed in litterbags and installed either on the soil surface or buried at a depth of 15 cm within a field. Prior to installation residues were cut into approximately 2 cm pieces and inserted into 10 × 20 cm litterbags with 50 µm openings (Ankom Scientific, Macedon, NY), at a rate of 6.5 g bag^−1^, which is equivalent to 3.25 Mg residue ha^−1^ on a dry matter basis, a rate higher than found on many smallholder farms but consistent with previously measured amounts of crop residue under intensive management (Agenor 1977). Care was exercised to include representative amounts of stem and leaf in each sample.

Litterbags were consecutively numbered, both inside and out, individually weighed, then placed in the field in a 0.5 m^2^ grid where both surface and subsurface placement of the appropriate residue occurred at each point. Surface-placed litterbags were affixed with ground staples. Litterbags from each residue and placement combination were collected from the field: 3, 7, 14, 28, 56 and 112 days after study initiation.

As the litterbags were collected, their contents were dried in a solar drier and sent back to Virginia Tech for analysis. Once dry, samples were weighed and mass loss determined as the difference of initial and final weight. Total C and N content in residues were determined by dry combustion (VarioMax CNS macro elemental analyzer, Elementar, Hanau, Germany).

### Statistical analysis

The experimental design was a split plot with a full factorial of four residue types and two placements. Main plot was placement and sub-plots were residue type. Regression was used to evaluate the effect of experimental factors on residue decomposition and C and N mineralization. Depending on the significance of the model, linear or nonlinear regression models available in SAS JMP Pro 11 (JMP 2013) were applied to least squares estimates for residue mass loss, and N, C, and C:N ratio measurements over time. The most appropriate regression model was chosen based on the significance of the model, lowest root mean square error (RMSE), highest R^2^, and reflects the higher-order model when these parameters were similar. Slopes of regressions or regression components for each residue and placement combination were compared to assess potential differences among treatments.

## Results and discussion

### Residue mass loss over time

**Fig. 2:**
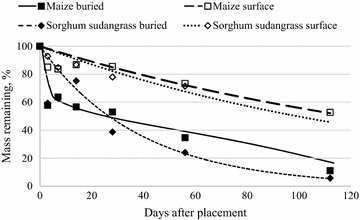
Buried and surface-placed maize and sorghum sudangrass residue mass loss (dry weight basis) over 112 days

Mass of both maize and sorghum sudangrass, either surface-placed or buried, decreased with time (Fig. 2). However decomposition of buried residues was faster (Table 3). Initially, the rate of mass loss was similar for both residue types when left on the surface. Both lost mass at a rate of approximately 2.2 % day^−1^ during the initial 7 days, and after that, both surface-placed grass residues continued to lose mass at a similar rate (Table 3). At 56 days, 73 and 71 % of the initial mass of surface-placed maize and sorghum sudangrass remained, respectively (Fig. 2). Buried residues, by comparison, decomposed more rapidly (Fig. 2, Table 4). At 112 days, less than 12 % of the original mass of maize residue remained and only 5 % of initial sorghum sudangrass mass. The difference in mass loss rate between the two residues is likely due to the lower initial C:N ratio in sorghum sudangrass compared to that of maize (Table 2). The relatively higher RMSE value for sorghum sudangrass also indicates greater variation in decomposition of this material compared to others. The initial (7 day) rate of decomposition was faster for buried maize residue than sorghum sudangrass but was similar afterwards (Table 3). Mass loss from day 0 to day 3 in buried residue was greater than 40 % for both grass residues. High initial mass loss rates within the first 3 days of the study may be partially due to favorable climatic factors. Rainfall occurred the day before litterbag installation and each of the first 3 days after installation. Daily average temperature (26.5 °C) was also favorable for decomposition. Although the surface-placed litterbags absorbed rainfall, it is likely that high daily temperatures resulted in more evaporation of water from these litterbags compared to below-ground litterbags. Higher moisture content and retention is often associated with increased rates of decomposition of residues in laboratory incubation and litterbag field studies (Douglas and Rickman 1992; Blagodatsky et al. 1998; Abera et al. 2014). Previous work has illustrated the combined role of residue C:N ratio, lignin and polyphenol content in determining the decomposition rate and nutrient mineralization rates of various residue types (Constantinides and Fownes 1994; Fox et al. 1990; Palm and Sanchez 1991; Palm et al. 2001). While not measured in this study, we believe that these components played a lesser role in our results because lignin and polyphenol content in the crop and legume residues studied typically have relatively low levels of both lignin and soluble polyphenols (Baijukya et al. 2006; Matta-Machado et al. 1994; Millar and Baggs 2004; Tian et al. 1992, 1995).

**Table 3:** Regression equations and statistics for residue mass loss, on a percent dry weight basis, over 112 days, where X = time in days after application

Residue species and placement	Equation	RMSE^a^	R^2^
Maize, buried	y = 66.65e^−0.0123x^ + 33.35e^−17.3890x^	6.214	0.974
Maize, surface	y = 100e^−0.006x^	5.152	0.939
Sorghum sudangrass, buried	y = 100e^−0.026x^	6.806	0.940
Sorghum sudangrass, surface	y = 100e^−0.007x^	9.476	0.665
Common bean, buried	y = 100e^−0.012x^	7.873	0.915
Common bean, surface	y = 100e^−0.004x^	6.777	0.609
Sunn hemp, buried	y = 52.30e^−0.0079x^ + 47.70e^−0.3136x^	3.742	0.990
Sunn hemp, surface	y = 63.44e^−0.0001x^ + 37.76e^−0.0306x^	7.932	0.863

^a^Root mean square error

**Table 4:** Regression equations and statistics for change in mass of residue N remaining over 112 days, where X = time in days after application

Residue species and placement	Equation	RMSE^a^	R^2^
Maize, buried	y = 0.03e^−0.0010x^ + 0.03e^−1.0601x^	0.007	0.910
Maize, surface	y = 0.36e^−0.0069x^ + 0.02e^−22.3816x^	0.003	0.976
Sorghum sudangrass, buried	y = −0.20 + 0.28e^−0.0022x^	0.012	0.829
Sorghum sudangrass, surface	y = 0.05e^−0.0055x^ + 0.02e^−0.6272x^	0.003	0.978
Common bean, buried	y = 0.02 + 0.02e^−0.0210x^	0.005	0.799
Common bean, surface	y = 0.03 + 0.02e^−0.0236x^	0.004	0.793
Sunn hemp, buried	y = 0.05e^−0.0087x^ + 0.07e^−0.3640x^	0.015	0.916
Sunn hemp, surface	y = 0.04 + 0.09e^−0.0561x^	0.007	0.972

^a^Root mean square error

**Fig. 3:**
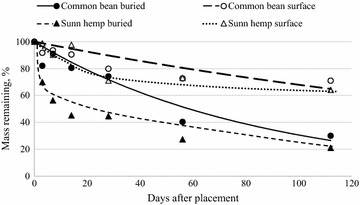
Buried and surface-placed common bean and sunn hemp residue mass loss (dry weight basis) over 112 days

Surface-placed sunn hemp lost mass at a faster rate than common bean over the first 28 days of the study (Fig. 3) but buried sunn hemp decomposition was similar to buried common bean. There was also greater variation in the decomposition of surface-placed sunn hemp, based on the higher RMSE (Table 3). This is likely a result of the relatively high C:N ration in the common bean residue (Table 2). The rate of buried sunn hemp residue decomposition was higher than common bean during the initial 56 days of the study, also likely due to the difference in C:N ratio of the two residues. Overall, decomposition rates were lower than those measured in similar work in Sub-Saharan Africa where haricot bean and pigeon pea residues lost 24–73 % of initial mass within the first 30 days of the study and common bean and sunn hemp lost 20–53 % (Abera et al. 2014). Ibewiro et al. (2000) compared mucuna [*Mucunapruriens* (L.) *DC.* var. *utilis* (Wright) Bruck)] and lablab (*Lablabpurpureus* L.) in a dryland savanna in Nigeria and also observed higher rates of mass loss (approximately 60 %) within the study’s first 30 days for both legumes regardless of placement. Likely due to advanced maturity and aging of the field crop residues, common bean C:N values were similar to those of the grass crops in our study (Table 2). Njunie et al. (2004) observed slower release of nutrients and decomposition of *Clitoria ternatea* (L.) and lablab in mature tissues when compared with younger cuttings of the same residues. The similar rates of decomposition of common bean and grassy crop residues likely stems from the relatively high C:N ratio of our common bean residues. However, since whole common bean plants are commonly removed from fields to facilitate dry-down and harvest and then piled or returned to fields, it is likely that our values are representative of what would occur with bean residue under current management.

The overall rates of surface versus buried residue decomposition in our study (Table 4) coincide with the findings from numerous others who also report that surface mass loss occurred at slower rates (Holland and Coleman 1987; Thomas and Asakawa 1993; Ibewiro et al. 2000; Thippayarugs et al. 2008; Abera et al. 2014). Greater contact with the microbial community in buried residues is theorized to be responsible for higher rates of decay in comparison to surface-placed residue. Additionally, the 50 µm mesh openings in the litterbags used in this work excluded surface meso- and macro-fauna from the decomposition process which contributed to the slower decomposition rates observed with surface-placed residues.

### Nitrogen mineralization

**Fig. 4:**
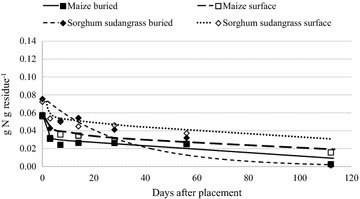
Net N mineralization from buried and surface-placed maize and sorghum sudangrass residue over 112 days

**Fig. 5:**
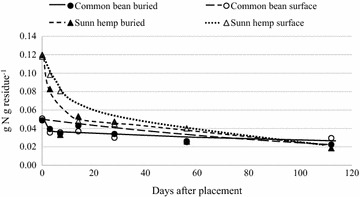
Net N mineralization from buried and surface-placed common bean and sunn hemp residue over 112 days

Nitrogen concentration decreased for all residues over 112 days, implying net mineralization of N (Figs. 4, 5). As expected, the highest amount of N mineralization occurred from the buried residues with the lowest C:N ratios (Table 2). Eighty-five percent of buried sunn hemp residue N was mineralized by 112 days, compared to 54 % of initial N of buried common bean (Fig. 5). Nitrogen mineralization from buried sorghum sudangrass and maize residues was greater than 90 % of the initial amount, though the absolute amount of N mineralized was less than that from the leguminous species (Figs. 4, 5). Overall, surface-placed residues with the lowest initial C:N ratios mineralized N at faster rates than those with higher initial ratios (Table 6). For instance, surface-placed sunn hemp residue N declined much more rapidly than common bean over the initial 14 days (Fig. 5). Previous research under similar conditions has reported residue N loss of greater than 54 % within 28 days (Ibewiro et al. 2000). These studies also documented a rapid initial N release over the first 60 days followed by a slower and curvilinear trend in N release from legumes. Net N mineralization in our study ranged from 10–60 % with an average of 43 % for buried residues and 35–60 % for surface- placed residue within the first 28 days. Buried sunn hemp N decreased by 78 % over 56 days (Fig. 5). Fosu et al. (2007) reported a 50 % decrease in N concentration of sunn hemp over a 70 days in a field study in Ghana. This rapid N release from sunn hemp under these conditions indicates promising decomposition kinetics for N from sunn hemp as a potential green manure in the Central Plateau.

### Carbon mineralization

**Fig. 6:**
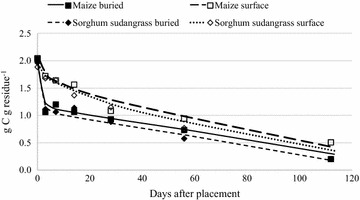
Net C mineralization from buried and surface-placed maize and sorghum sudangrass residue over 112 days

**Fig. 7:**
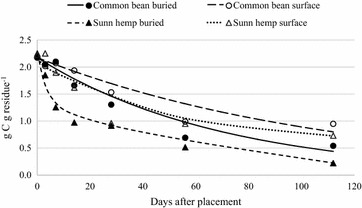
Net C mineralization from buried and surface-placed common bean and sunn hemp residue over 112 days

Retention of crop residue on or near the soil surface has been shown to reduce residue C loss and increase SOC over time (Lal 2004). We observed average residue C loss rates of 68 % for surface-placed residue and 86 % for buried residue (Figs. 6, 7). This decrease in C concentration for buried residues is similar to what was observed for mass loss because soil C mineralizes faster when in greater contact with soil microbial biomass (Figs. 6, 7). Similar trends have been demonstrated throughout tropical and temperate systems worldwide (e.g. Ono et al. 2009).

The N limited soil system where the litterbags were deployed may serve as explanation for the minimal release of C from surface-placed residue in comparison to buried residues. Sunn hemp exhibited higher C loss rates than common bean during the first 14 days regardless of placement. However, maize C mineralization exceeded that from sorghum sudangrass regardless of placement (Table 5). The C loss of maize was similar to sunn hemp and sorghum sudangrass throughout the study (Figs. 5, 6) despite the higher C:N ratio of maize (Table 2). Thomas and Asakawa (1993) tested decomposition rates of six legumes and four cereals in Columbia and also reported similar C losses for grasses and three of the legumes studied [*Centrosema acutifolium* Benth.*, Pueraria phaseoloides* Benth. and *Stylosantes guianensis* (Aubl. Sw.)]. They concluded that mineralization potential was highly species-dependent and this, along with residue maturity and aging, may help explain our results as well.

**Table 5:** Regression equations and statistics for change in mass of residue C remaining over 112 days, where X = time in days after application

Residue species and placement	Equation	RMSE^a^	R^2^
Maize, buried	y = 0.03e^−0.0010x^ + 0.03e^−1.0601x^	0.007	0.910
Maize, surface	y = 0.36e^−0.0069x^ + 0.02e^−22.3816x^	0.003	0.976
Sorghum sudangrass, buried	y = −0.20 + 0.28e^−0.0022x^	0.012	0.829
Sorghum sudangrass, surface	y = 0.05e^−0.0055x^ + 0.02e^−0.6272x^	0.003	0.978
Common bean, buried	y = 0.02 + 0.02e^−0.0210x^	0.005	0.799
Common bean, surface	y = 0.03 + 0.02e^−0.0236x^	0.004	0.793
Sunn hemp, buried	y = 0.05e^−0.0087x^ + 0.07e^−0.3640x^	0.015	0.916
Sunn hemp, surface	y = 0.04 + 0.09e^−0.0561x^	0.007	0.972

^a^Root mean square error

### Carbon:nitrogen ratio

Maize C:N ratio was initially near 54:1 and decreased at the same rate for both buried and surface-placed residue through the first 28 days, after which the C:N ratio of the buried maize decreased at a faster rate than at the surface (Table 6). Sorghum sudangrass C:N ratio was initially 31:1 (Table 2) and so the rate of decrease was lower than for maize (Table 6). At 56 days, sorghum sudangrass C:N ratio was near 20:1 for both surface and sub-surface-placed residue.

**Table 6:** Regression equations and statistics for change in residue C:N ratio over 112 days, where X = time in days after application

Residue species and placement	Equation	RMSE^a^	R^2^
Maize, buried	y = 35.04e^−0.0040x^ + 20.94e^−0.0579x^	2.184	0.985
Maize, surface	y = 40.56e^−0.0020x^ + 14.66e^−0.0586x^	2.335	0.961
Sorghum sudangrass, buried	y = 21.44e^−0.0021x^ + 10.03e^−0.3034x^	1.209	0.969
Sorghum sudangrass, surface	y = 31.27 − 0.2126x	1.411	0.944
Common bean, buried	y = 21.88 + 32.50e^−0.0283x^	8.769	0.734
Common bean, surface	y = 17.26 + 41.10e^−0.0093x^	2.235	0.966
Sunn hemp, buried	y = 11.04e^−0.0623x^ + 21.85e^−0.0048x^	7.306	0.670
Sunn hemp, surface	y = 20.26e^−0.0006x^ + 9.98e^−0.3873x^	1.418	0.939

^a^Root mean square error

**Fig. 8:**
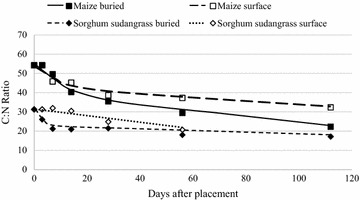
Buried and surface-placed maize and sorghum sudangrass residue C:N ratio over 112 days

**Fig. 9:**
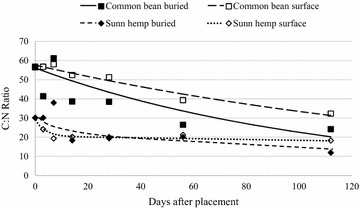
Buried and surface common bean and sunn hemp residue C:N ratio over 112 days

Initial common bean C:N ratio was similar to that of maize (Figs. 8, 9) and the change in C:N ratio was similar as well. Burying bean residue resulted in a more rapid and overall greater decrease in C:N ratio compared to placing it on the surface (Table 6). Sunn hemp, the residue with the lowest C:N in our litterbag decomposition study exhibited the fastest decomposition rates when buried (Table 6) Abera et al. (2014) reported similar outcomes for pigeon pea and haricot bean, as pigeon pea had a lower C:N ratio and was also found to release N more rapidly. However, when sunn hemp residue remained on the soil surface, C:N ratio remained near 20:1 from day 14 through the duration of the study (Fig. 9). More rapid decrease in C:N ratio over time for residues with low C:N ratios has been substantiated by numerous other researchers in laboratory and litterbag field analysis (Blagodatsky et al. 1998; Fosu et al. 2007; Zhang et al. 2008; Abera et al. 2014). Similar to what was observed for mass loss and N mineralization of these residue samples, C:N ratio of most residues decreased over 112 days regardless of placement or residue quality.

## Conclusions

Initial residue C:N affected C and N mineralization rates and mass loss for the residues we tested, with higher N concentration and narrower C:N ratio resulting in more rapid mass loss. This was expected based on previous research, and indicates that a legume with high N concentration is the ideal candidate cover crop for supplying N to a succeeding food crop. In our work, sunn hemp contained the highest N concentrations and released the most N to the system in the shortest amount time. Overall, burying residue resulted in much faster decomposition and N mineralization over the course of our study. In N-poor systems such as those in the Central Plateau, if the goal of a legume cover crop is to supply N to the succeeding crop, incorporating that cover crop into the soil with tillage will hasten decomposition and nutrient release. However, using tillage will likely increase soil erosion and nutrient depletion compared to no-tillage. We observed much slower overall mass loss when any of the residue types was left on the soil surface. This indicates that all would be somewhat effective in protecting soil from erosive effects of rainfall. However, those residues with high C:N ratio (grasses and weathered legume residue) will likely offer protection for a longer time. The continued utilization of legumes and beans in relay and intercrops along with the introduction of short-season cover crops could help to rebuild soil structure and increase SOM. In order to achieve both goals of keeping soil covered and increasing nutrient supply to other crops in the rotation it may be necessary to employ a combination of cover crops at different times in the rotations and to utilize cover crops over an extended period of time to improve the overall N status of the system.
